# Improving natural red pigment production by *Streptomyces phaeolivaceus* strain GH27 for functionalization of textiles with in silico ADME prediction

**DOI:** 10.1186/s12866-024-03697-4

**Published:** 2025-01-13

**Authors:** Gehad H. El Sayed, Mohamed Fadel, Rasha Fouad, Hend M. Ahmed, Ahmed A. Hamed

**Affiliations:** 1 Microbial Chemistry Department, Biotechnology Research Institute, National Research Center, Dokki, Giza, Egypt; 2 Medicinal and Aromatic Plants Research Department, National Research Centre, Dokki, Giza, Egypt; 3 Dyeing, Printing and Intermediate Auxilaries Department, Textile Research and Technology Institute, National Research Center, Dokki, Giza, Egypt

**Keywords:** Actinomycetes, Red pigment, Optimization, Production, Textiles, Antimicrobial fabrics, Molecular docking

## Abstract

The red pigment was recovered from the *S. phaeolivaceus* GH27 isolate, which was molecularly identified using 16S rRNA gene sequencing and submitted to GenBank as OQ145635.1. The ideal growth conditions included 1% (w/v) starch, diammonium citrate, dibasic sodium phosphate, 5% (v/v) inoculum, *p*H 8, a rotation speed of 150 rpm, a temperature of 37 °C, and an incubation period of 9 days. Using ethanol as a solvent, the red pigment was effectively recovered. Data indicates that pigment content remained steady at 40 and 50 °C. Heating the pigment extract to 60, 70, 80, 90, and 100 °C for one hour results in pigment retention of 98%, 96.5%, 95.5%, 94.6%, and 92.6% of its pigment density, respectively. Studies indicate that the pigment extracts exhibited optimal stability at alkaline *p*H levels. The findings demonstrate that the red pigment extract has a peak absorbance range of 280–340 nm, with a λmax of 300 nm. GC/MS analysis revealed that the primary components of the pigment extract were linolenic acid methyl ester and oleic acid methyl ester, constituting 26.41% and 25.25%, respectively. Fabrics dyed with extracted red pigment exhibit excellent fastness when using the comprehensive green method. In comparison to conventional and nanotechnological attributes, printed samples exhibit significant color strength without environmental repercussions. The treatment of cotton, wool, and polyester samples suppressed pathogen growth to differing extents. Polyester had the most important inhibitory effects on *Staphylococcus aureus* (50.03%) and *Bacillus cereus* (39.49%). The ADME physicochemical properties of the predominant medication were assessed, together with its bioavailability. The radar plot demonstrated ideal parameters for size, polarity, lipophilicity, solubility, and saturation, excluding flexibility. It exhibited intermediate synthetic accessibility, exceptional permeability and absorption, elevated gastrointestinal absorption, and blood–brain barrier penetration; nonetheless, it did not adhere to the medicinal chemistry rule of three.

**Graphical Abstract:**

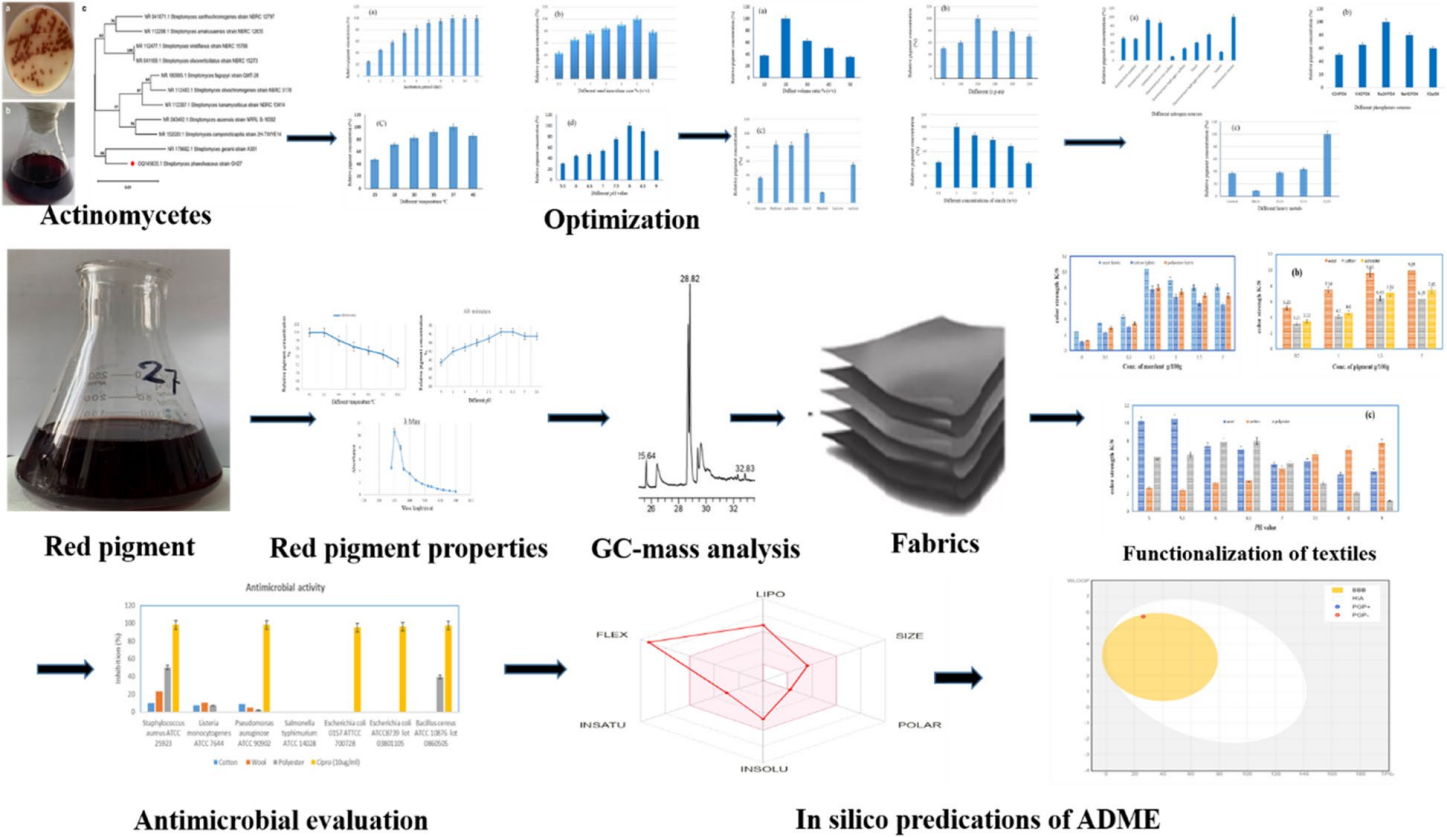

**Supplementary Information:**

The online version contains supplementary material available at 10.1186/s12866-024-03697-4.

## Introduction

Recently, there has been a renewed focus on bacterial Pigments are the major sources of colors and are also key components in manufacturing paint [1]. Numerous artificial colors used in the production of food, dyes, cosmetics, and pharmaceuticals have a number of dangerous side effects, such as cancer, tumors, allergies, and serious vital organ damage [2]. Consequently, because of their toxicological troubles, many synthetic colors are now prohibited. Interest in creating colors from natural sources is growing as people become more conscious of consumer safety and the harmful consequences of synthetic colors [2]. Among the earliest civilizations were those of ancient Egypt, China, and India to use natural plants, insects, and minerals to produce paint, dye textiles, color food, color the body during ceremonies of religion, and other uses for pigments that have a long history [3, 4]. The bio-colors are used in the food, textile, and pharmaceutical industries as additives, color intensifiers, antioxidants, etc. [5, 6]. Increased environmental consciousness has made the use of natural dyes in textiles that are both safe to use, not allergic, and environmentally friendly extremely important [7]. Microorganisms produce biopigments that have been preferred over plant-based ones due to their stability [8] and year-round culture availability [5, 9]. It was observed that these microorganisms had the ability to grow and produce a wide variety of bio-pigments such as prodigiosin, carotenoids, flavones, quinines, melanins, and monascins [10, 11]. Color-producing microorganisms (bacteria, yeasts, and fungi) are widespread in nature. One of the newer areas of study showing the potential for numerous industrial uses is bacterial pigment synthesis [7, 12]. The benefits of using bacteria to produce pigment include fast and easy growth in cheap culture media, independent of weather, and faster fermentation for bulk production [13]. More than 90% of actinomycetes members had been isolated from the soil and widely used in many industrial, agricultural, and medical applications [14, 15]. Actinomycetes can produce numerous metabolites like chemicals that are biologically active [14–17] and different pigments with many colors, such as green, yellow, brown, red, violet, and black. The substantial patterns of pigment diversity and strains of potential biotechnological interest, such as secondary metabolite-derived pigments as natural yellow food colorants, have been identified in different bacterial strain collections; some of the pigment producers were actinomycetes. The pigment-producing actinomycetes can primarily be used beneficially as probiotics for marine animals and plant growth-promoting microbes and can efficiently degrade some recalcitrant pollutants. These pigmented actinomycetes could thus have environmentally helpful applications, as well as biopharmaceutical and food colorant applications [14, 18]. Actinomycetes produce pigments in response to changes in the *p*H of their growth media as well as single carbon and nitrogen sources. To create high-end products, fermentation conditions, metabolic engineering, strain enhancement, and simple extraction methods are required [19, 20]. The use of natural pigments extracted from *S. phaeolivaceus* strain GH27 in printing on various fabrics, including wool, cotton, and polyester, presents an interesting opportunity to study their potential antimicrobial effects on textiles [6, 21]. This research would be valuable in addressing the growing interest in functional textiles that offer added benefits such as antibacterial or antifungal properties. Furthermore, it could open up new possibilities for utilizing natural resources in textile production while simultaneously enhancing the performance and functionality of fabrics. The findings from such a study could have implications for diverse sectors, including healthcare, fashion, and outdoor apparel, where antimicrobial textiles are increasingly sought after for hygiene and sustainability reasons.

## Materials and methods

### Sample collection

Rhizosphere soil samples were procured from a depth of 8–10 cm using a sterile spatula. The samples were brought to the laboratory and processed further immediately.

### Pre-treatment of the soil sample

The soil samples were pretreated in order to reduce the proportion of other microorganisms that differ from Actinomycetes. It had dried for about 5–10 min at 50–60 °C [22].

### Isolation and screening of actinomycetes

According to El Sayed et al. (2023), isolation of actinomycetes occurs [3]. Starch nitrate agar medium is used for isolation. The medium contained (g/L): starch (20), KNO_3_ (2.0), K_2_HPO_4_ (1.0), CaCO_3_ (3.0), MgSO_4_ (0.5), NaCl (0.5), FeSO_4_ (0.01), and agar (20) at *p*H 7.8. All the plates were screened for actinomycetes colonies based on morphology and pigmentation. The actinomycete colony that showed significant coloration was subcultured onto a fresh medium by streaking until a pure culture was obtained.

### Molecular identification of bacterial isolate

#### Genomic DNA extraction

In order to produce heavy growth that could be used for DNA extraction, a single colony of *S. phaeolivaceus* strain GH27 was picked up, streaked on starch nitrate agar plates, and incubated at 28 °C for five days. Thermo ScientificTM's GeneJET Genomic DNA Purification Kit (Waltham, MA, USA) was used to extract genomic DNA from bacterial culture. The 16S rRNA gene was amplified using universal bacterial primers, specifically the 8F forward primer (5′AGAGTTTGATCMTGGCTCAG-3′) and the 1429R reverse primer (5′-TACGGYTACCTTGTTACGACTT-3′).

### PCR amplification and sequencing of the amplified PCR product

PCR involved an initial denaturation at 95 °C for 3 min, followed by 35 cycles of denaturation at 95 °C for 30 s, annealing at 50 °C for 30 s, and elongation at 72 °C for 1 min. The final extension was carried out for an additional 10 min at 72 °C using the BioRad T100 Thermal Cycler. The amplified fragment was examined on a 1.2% agarose gel stained with ethidium bromide. PCR products have been purified using the GeneJET Gel Extraction Kit from Thermo Scientific™ (Waltham, MA, USA). They have been sent to Macrogen Inc. (Amsterdam, the Netherlands) for 16S rRNA sequencing by using the high-throughput Applied Biosystems 3730XL sequencer.

### Phylogenetic analysis

Software called BioEdit 7.1.10 was used to edit the sequence [23]. The picked-up sequences were compared to those in the GenBank database (http://www.ncbi.nlm.nih.gov/blast) in order to identify similar species. A phylogenetic tree was built using a set of 16S rRNA genes from related species that were taken from the GenBank database. Multiple sequence alignments were performed in MEGA11 [24] using the MUSCLE [25] method. The neighbor-joining approach [26] with a 1000-bootstrap run [27] was used to infer the evolutionary history, and the Jukes-Cantor method [28] was used to calculate evolutionary distances.

### Inoculum seed preparation

Five-day-old actinomycetes culture slants were obtained by adding 10 ml of sterilized water, where the growth was crushed with a culture loop. A 1.5% spore suspension was used to inoculate 250 ml conical flasks containing 50 ml of pigment production medium (g/L). Starch, 20, KNO_3_, 2.0, K_2_HPO_4_, 1.0, MgSO_4_, 0.5, NaCl, 0.5, CaCO_3_, 3.0, FeSO_4_, 0.01, *p*H, 7.8. Afterward, incubation was done in a rotary shaker at 150 rpm for 5 days at 28 °C.

### Standardization of culture conditions for optimum pigment production

The effect of various cultural conditions was studied, like different incubation periods (3–12 days), different inoculum sizes (2–8%), different incubation temperatures (25, 28, 31, 34, 37, and 40 ºC), *p*H (6, 6.5, 7, 7.5, 8, 8.5, and 9), different broth medium volumes (10–50%) (v/v) in a 250 ml conical flask, different shaking speeds, different carbon sources, different carbon source concentrations, different nitrogen sources, different phosphorous sources, and different heavy metal ions (Zn^++^, Mn^++^, Cu^++^, and Cr^+++^). The pigment production was studied by inoculating a bacterial suspension of the *S.* *phaeolivaceus* GH27 strain.

### Extraction of the pigment

At the end of the fermentation period, the whole biomass of the culture is filtered off through a Whatman No. 1 filter paper. The filtrate was then centrifuged at 10,000 rpm for 10 min. Supernatants were considered the source of extracellular pigment. Extraction of the pigment was done according to [1, 29]. Briefly, pigment extraction was conducted using ethanol as an extracting solvent using liquid–liquid extraction (1:1). The recovered extract containing the pigment was vacuum-dried at lower pressure. The thermal stability of red pigment was determined according to [30]. The extracted red pigment was subjected to various temperatures for 60 min. Based on the absorbance measured for the pigment extract before and after heat treatment, the retention of pigment was calculated. The pigment sample was exposed to a controlled range of temperatures (40 °C to 100 °C). The effect of pH variation on the stability of red pigment was studied as described by [31]. A UV–visible spectrophotometer assay was carried out to determine the range of λmax (Parmar and Singh 2018) [29].

### Characterization of the pigment by GC/MS analysis

El-Sayed et al. (2023) carried out sample derivatization. The constitutive metabolites were identified by matching their mass spectra and other MS parameters with the corresponding ones of the WILEY 09 and NIST 14 mass spectral library databases [32].

### Printing of cotton, wool, and polyester fabrics with extracted red pigment

To create a printing paste, combine 2 g of synthetic thickener, a binder ranging from 5 to 20 g, 4 g of urea, and 0.5 g of sodium dihydrogen phosphate. Incorporate 2 g of dyes into the mixture, then add enough water to reach a total weight of 100 g. The printing and thermofixation process was used to fix all of the printed samples for three minutes at 180 °C. The samples were then wholly rinsed with cold water, hot water treated at 60 °C using two g/l nonionic detergent, hot water rinsed, and finally, cold water rinsed. In order to estimate the color strength, the samples were evaluated after drying. In all these tests, the goal is to ensure that the dyed fabrics maintain their appearance and do not transfer or lose color under real-world conditions. Using these standardized methods helps ensure consistency and reliability in color fastness evaluation.

The three fabrics were as follows:

1. Cotton: El-Mahala El-Kubra Company provided the cotton in Egypt. The cotton material was entirely washed with cold tap water and then dried at ambient temperature after being scoured in an aqueous solution in a liquor ratio of 1:50 containing two g/L nonionic laundry detergent (hospital, Clariant) at 50 °C for 30 min to remove the harmful substances.
2. Wool: the materials were thoroughly cleaned with water and allowed to air dry at room temperature after being subjected to a solution containing two g/L of nonionic detergent (TERGITOLTM NP-9 Surfactant) at 50 °C for 30 min.
3. Polyester material was utilized. A mixture of 3 (g/L) sodium carbonate, 0.5 (g/L) wetting agent, and one (g/L) synthetic detergent was used to scrub the fabric for 10 min.

### Chemicals

Laboratory-grade chemicals are utilize tannic acid, urea, nonionic detergent, and ammonium persulfate (NH_4_)_2_S_2_O_8_ as thermal initiators. We purchased thermal curing thickeners and binders from Sigma Aldrich.

### Measurements

#### Color assessment (K/S)

The Hunter Lab Ultra Scan PRO is used to comprehensively analyze the color properties, and the Data Color SF 600 Plus is used to measure the color strength (K/S) of the dyed samples precisely. This combination helps achieve accurate and reliable color evaluations.

#### Fastness properties

You're detailing the methods used to evaluate the color fastness of dyed fabrics, which is crucial for determining how well the color will hold up under different conditions. Here's a breakdown of the tests and rating scales you mentioned:

1. AATCC Test Method 16–2001 (Color Fastness to Light): This test evaluates how well a fabric's color withstands exposure to light. It typically uses a xenon arc lamp to simulate sunlight. The rating scale ranges from 1 (very poor) to 8 (outstanding), with higher numbers indicating better resistance to light fading.
2. AATCC Test Method 61–2001 (Color Fastness to Laundering): This test assesses how well a fabric's color holds up through repeated laundering. It's often evaluated in terms of color change and staining on adjacent fabrics. The rating scale for color change due to washing ranges from 1 (very poor) to 5 (excellent).
3. AATCC Test Method 8–2001 (Color Fastness to Rubbing): This test measures how well the color of a fabric resists rubbing or abrasion. It's assessed by rubbing the fabric against a white cloth and evaluating the amount of color transfer. The rating scale is similar to the laundering test, from 1 (very poor) to 5 (excellent).
4. Color Fastness to Perspiration: This evaluates how well the fabric's color withstands exposure to sweat, which can affect color stability. The rating scale for perspiration fastness is generally similar to the washing fastness scale, ranging from 1 (very poor) to 5 (excellent).

In all these tests, the goal is to ensure that the dyed fabrics maintain their appearance and do not transfer or lose color under real-world conditions. Using these standardized methods helps ensure consistency and reliability in color fastness evaluation.

#### Preparation of printing paste

**Table:** 

The printing paste recipe:		
Synthetic thickener	2	g
Binder	(5–20)	g
Urea	4	g
Sodium dihydrogen phosphate	0.5	g
Dyes	2	g
Water	X	g
	100	g

The thermalization process fixed all of the printed samples for three minutes at 180 °C. The samples were then completely rinsed with cold water, hot water treated at 60 °C using 2 g/l nonionic detergent, hot water rinsed, and cold water rinsed. The samples were evaluated after drying to estimate the color strength.

#### Antimicrobial activity

Antimicrobial screening was carried out against a group of test microbes [33]. In all, ten μL of compounds (250 ug/ml) have been added to 180 μL of culture medium, i.e., lysogeny broth for bacteria, followed by the addition of 10 μL of bacterial or fungal suspension at the log phase. After the plates had been incubated at 37 °C overnight, by using a Spectrostar Nano Microplate Reader (BMG Labtech GmbH, Allmendgrun, Germany), the absorbance had been measured at OD 600. Eight serial dilutions (250, 125, 62.5, 31.25, 15.63, 7.81, 3.90, and 1.95 µg/mL) were first prepared, and, according to the obtained results, further dilutions bracketing the lowest concentration with no noticeable growth of bacteria or fungi were tested in steps of 1 µg/mL. MICs are defined as the mean of the lowest concentrations at which no noticeable bacterial or fungal growth occurred, as assessed by three separate experiments. Ampicillin was used as a positive control.

## Results and discussion

### Isolation

**Fig. 1:**
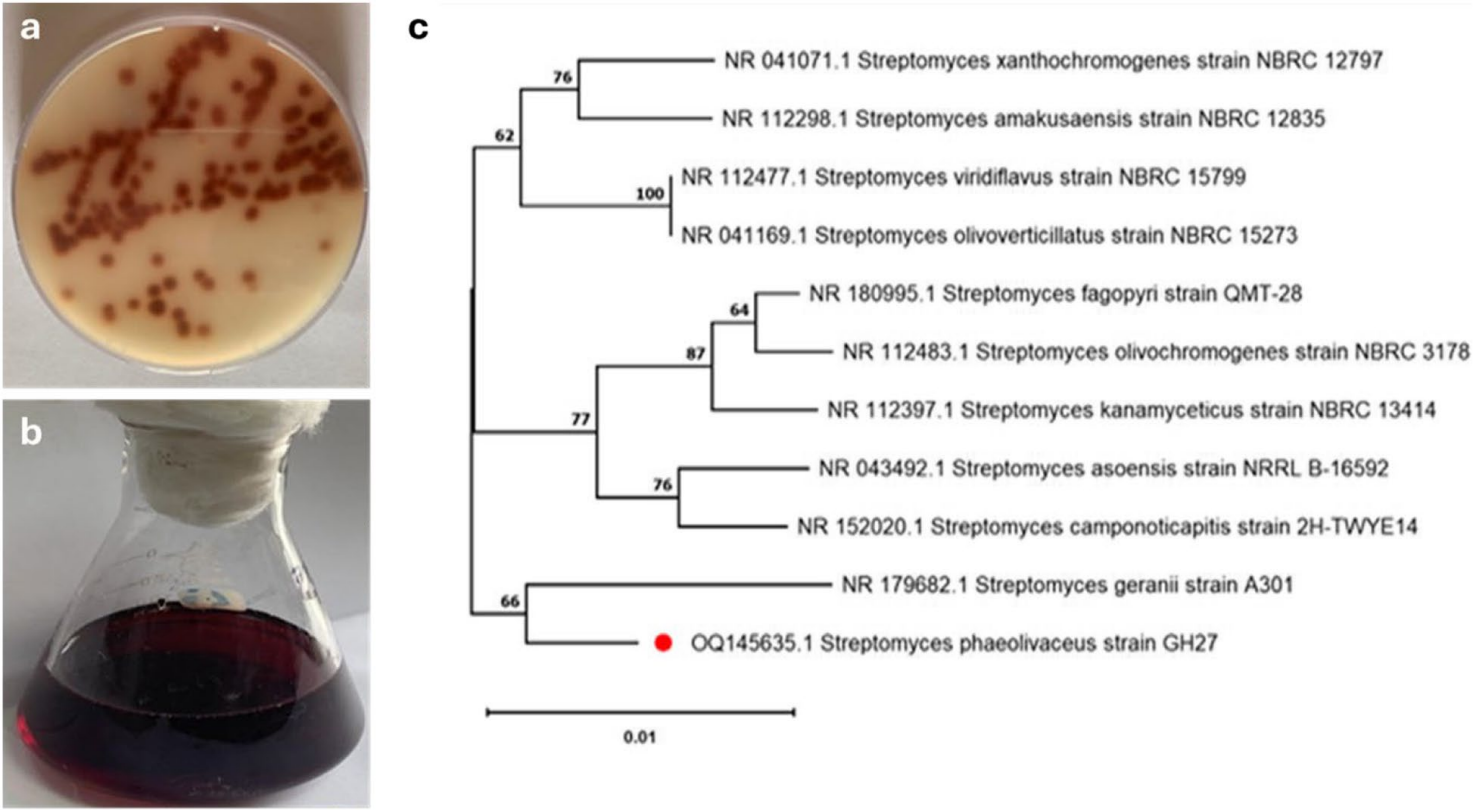
**a** Diffusible red pigment produced by S. *phaeolivaceus* GH27 on solid media. **b** Diffusible red pigment in the media produced by S. *phaeolivaceus* GH27. **c** Phylogenetic tree of *S. phaeolivaceus* GH27 based on 16S rRNA gene sequences

Actinomycetes are a group of prokaryotic microorganisms’ gram-positive filamentous bacteria. A number of 135 Streptomycetes colonies were randomly chosen and isolated from different soil sources in Egypt. From the isolated Streptomycetes isolates, the *S.* *phaeolivaceus* GH27 strain was selected as a sharp red pigment producer (Fig. 1a and b).

### Molecular identification of the actinomycetes isolate

The NCBI Gene Bank database accepted the *Streptomyces* isolate identified in the current study and registered it with accession number OQ145635.1. The analysis of the *S. phaeolivaceus* GH27 phylogeny was done using the sequence of the ITS region. By comparing this isolate to the ITS recorded sequences that were retrieved from NCBI, it was possible to identify the isolate at the genus and species levels. The obtained phylogenetic tree showed that the GH27 isolate belongs to the genus *Streptomyces* and had a tight relationship with each other. The cluster showed a close relationship in the same clade, with a bootstrap value of 100%, as presented in the cluster (Fig. 1c).

### Optimum culture conditions for highest pigment production

In basic terms, pigment synthesis is correlated with cell growth. It is impacted by environmental factors, microbiological characteristics (such as spore, seed, and inoculum ages), and nutritional factors (such as carbon and nitrogen sources). Several important environmental variables need to be studied and improved [4].

### Effect of incubation period on the red pigment production

**Fig. 2:**
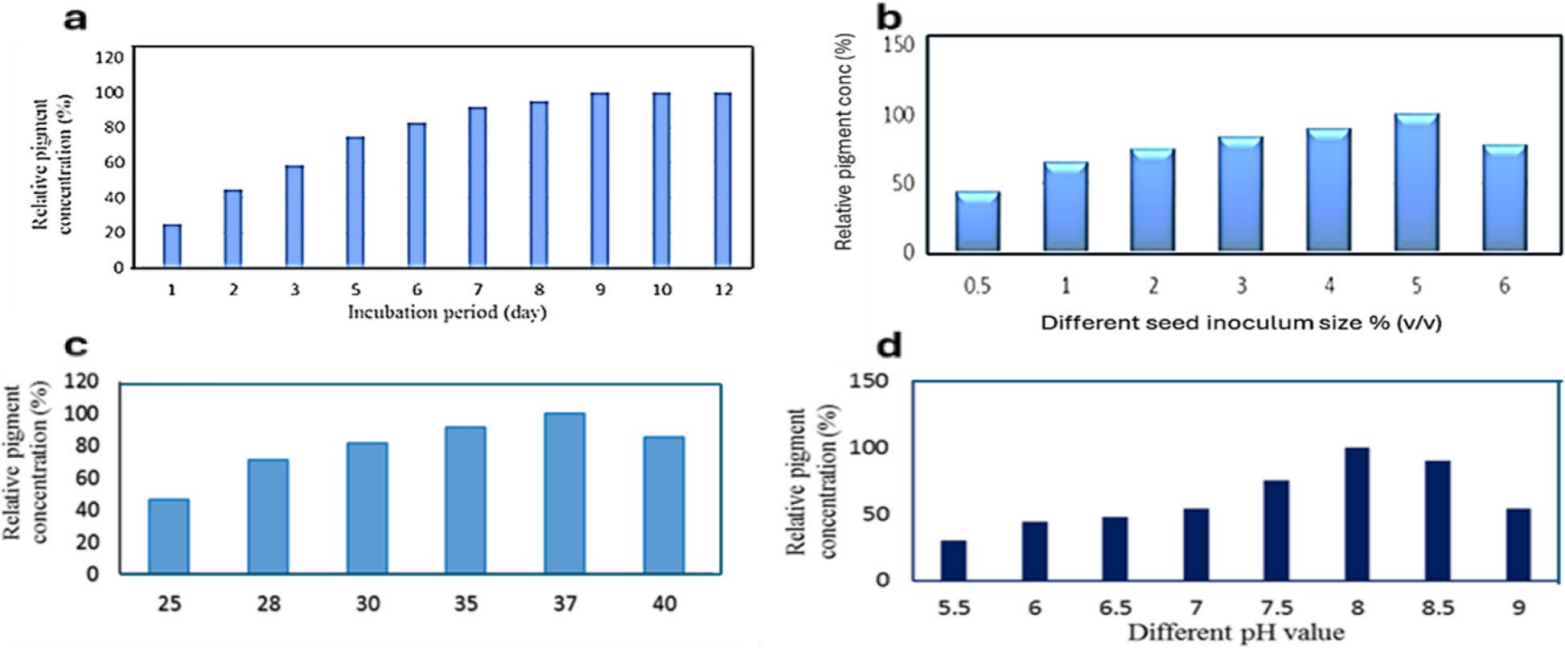
**a** Effect of incubation period, **b** Effect of inoculums size (v/v), **c** Effect of incubation temperature and **d** Effect of different initial *p*H value on the red pigment production by *S. phaeolivaceus* GH27

According to earlier studies, every strain has a different ideal incubation period for producing the highest level of pigment. As alongside Fig. 2a, the concentration of red pigment secretion rose as the incubation period was extended until the ninth day; at that point, it became constant. Elattaapy and Selim (2020) noted their maximum pigment synthesis after 8 days [34]. After 12 days of incubation, Santos-Ebinuma et al. (2013) produced the most incredible pigment [35]. However, after 24 days of incubation, Chadni et al. (2017) had the highest pigment concentration at incubation of 24 days [36]. However, a number of authors reported shortened incubation periods.

### Effect of inoculum size (v/v) on the red pigment production

When pigment produced by microorganisms was studied, the percentage of inoculums was taken into consideration [37, 38]. In comparison to other smaller or higher sizes of inoculums, Fig. 2b demonstrated that the inoculation medium containing 5% (v/v) inoculums recorded the maximum red pigment synthesis by *S. phaeolivaceus* GH27. It was stated that more significant inoculum sizes result in increased biomass and decreased pigment production because they prevent the increased biomass from utilizing the culture medium's vital nutrients [39]. El-Attaapy and Selim (2020) observed the most incredible pigment synthesis at 106/ml spore concentration [34].

### Effect of incubation temperature on the red pigment production

A key factor for growth and the metabolism of nutrients is temperature. Because the enzyme activity for pigment synthesis appears to be improved at this temperature, the maximum pigment production was obtained at that optimal temperature. The results demonstrate that the *S. phaeolivaceus* GH27 strain produced the reddest pigment when the incubation temperature was 37 °C (Fig. 2c). According to El Sayed et al. (2023), 37 °C is the ideal temperature for producing pigment [3]. Joshi et al. (2011) found that the best temperature for *Sarcina sp.* to make the most significant pigment secretion was 35 °C [40]. Zahan et al. (2020) found that the ideal temperature for pigment production was 30 °C [41].

### Determination of suitable initial pH value for red pigment production

Despite the growth rate not being affected in the same way, the results show that the initial *p*H has a substantial impact on pigment synthesis [34]. The pigment produced by the same organisms may have a different color due to changes in the *p*H of the fermentation medium. The findings presented in Fig. 2d demonstrated that the *S. phaeolivaceus* GH27 strain reached its peak pigment production concentration at *p*H 8, and that variations in *p*H levels have a significant impact on the formation of red pigment. The results indicate that the initial *p*H has a significant effect on pigment synthesis, even though the growth rate is not changed in the same way [34]. El Sayed et al. (2023) reported the same findings [3].

### The effect of medium volume on the red pigment production

**Fig. 3:**
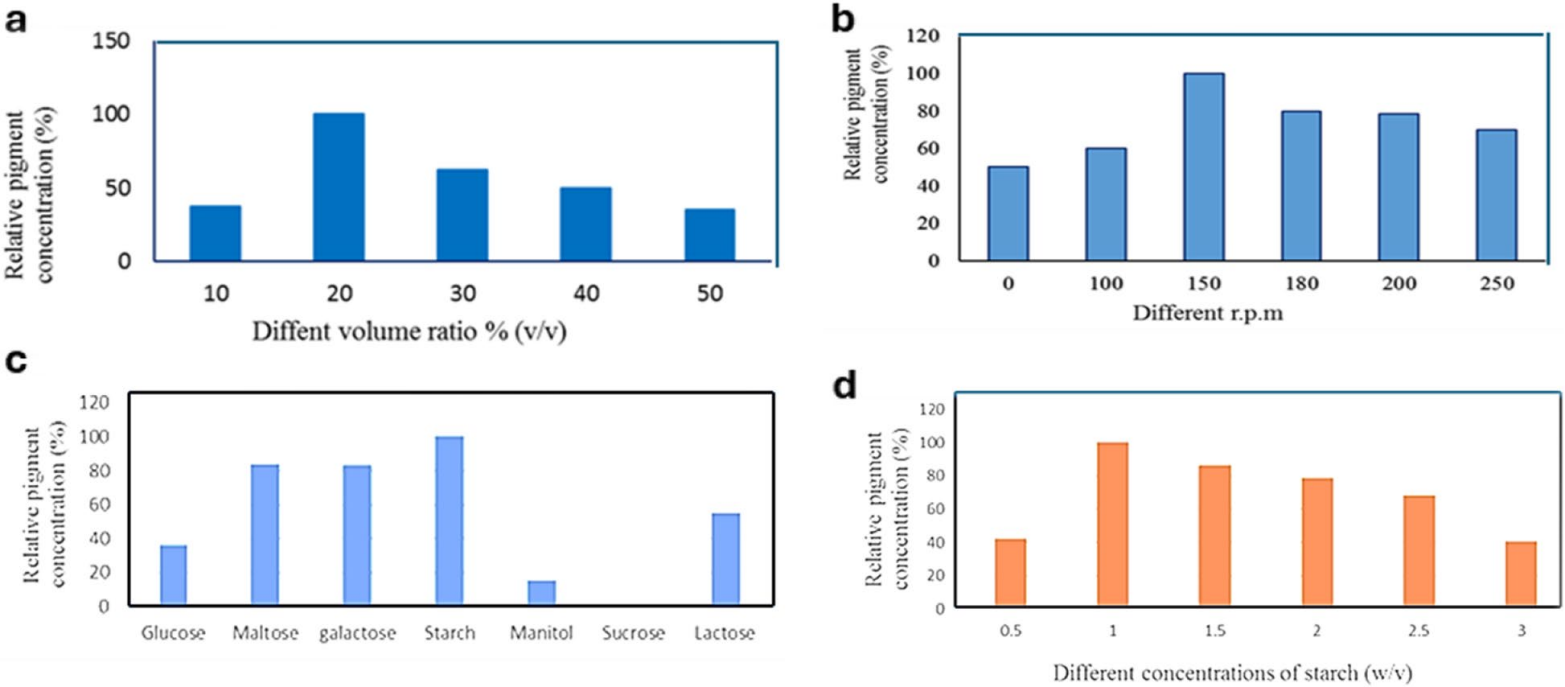
**a** Effect of culture volume % (v/v), **b** Effect of different rotation speed, **c** Effect of different carbon source and **d** Effect of starch concentration on the red pigment production by *S. phaeolivaceus* GH27

The volume of the culture has an impact on the amount of oxygen accessible for the growth of the organism, the use of nutrients in the culture, and ultimately the production of pigment. According to the study's findings, the highest concentration of red pigment was obtained when 20% (v/v) of the growing medium was used in 250 ml conical flasks (Fig. 3a).

### Effect of agitation (shaker speed) on the red pigment production

The maximum bacterial growth and red pigment content can be observed with shaken cultures at 150 r.p.m. (Fig. 3b). This could be due to the aeration, which improved the transport of oxygen and substrates and hence improved the growth and functionality of microbial cells [42, 43].

### Effect of different carbon sources on the red pigment production

Results indicated that insoluble starch was a more stimulating carbon source for the red pigment secretion in the growth medium (Fig. 3c). While maltose, galactose, and lactose could produce about 55–84% of red pigment as compared to starch. On the other hand, mannitol and glucose give a low amount of red pigment (15 to 36%) comparable to starch. Sucrose was unable to stimulate the red pigment production by *S. phaeolivaceus* GH27. In a similar way, starch was efficiently used for *Paecilomyces sinclairii's* synthesis of red pigment [44, 45]. Actiomycetes are known to use starch more frequently as a carbon source for pigment formation, but *Sarcina sp.* and *Exiguobacterium aurantiacum* FH have been shown to use glucose and fructose as more appropriate carbon sources [40, 46].

### Effect of starch concentration on the red pigment production

The growing medium with a 1% (w/v) starch concentration produced the highest red pigment concentration. The synthesis of red pigment was adversely impacted by the rising starch concentration (Fig. 3d). The best green pigment synthesis was achieved by El Sayed et al. (2023) using 2.5% (w/v) starch [3].

### Effect of inorganic nitrogen sources on red pigment production

**Fig. 4:**
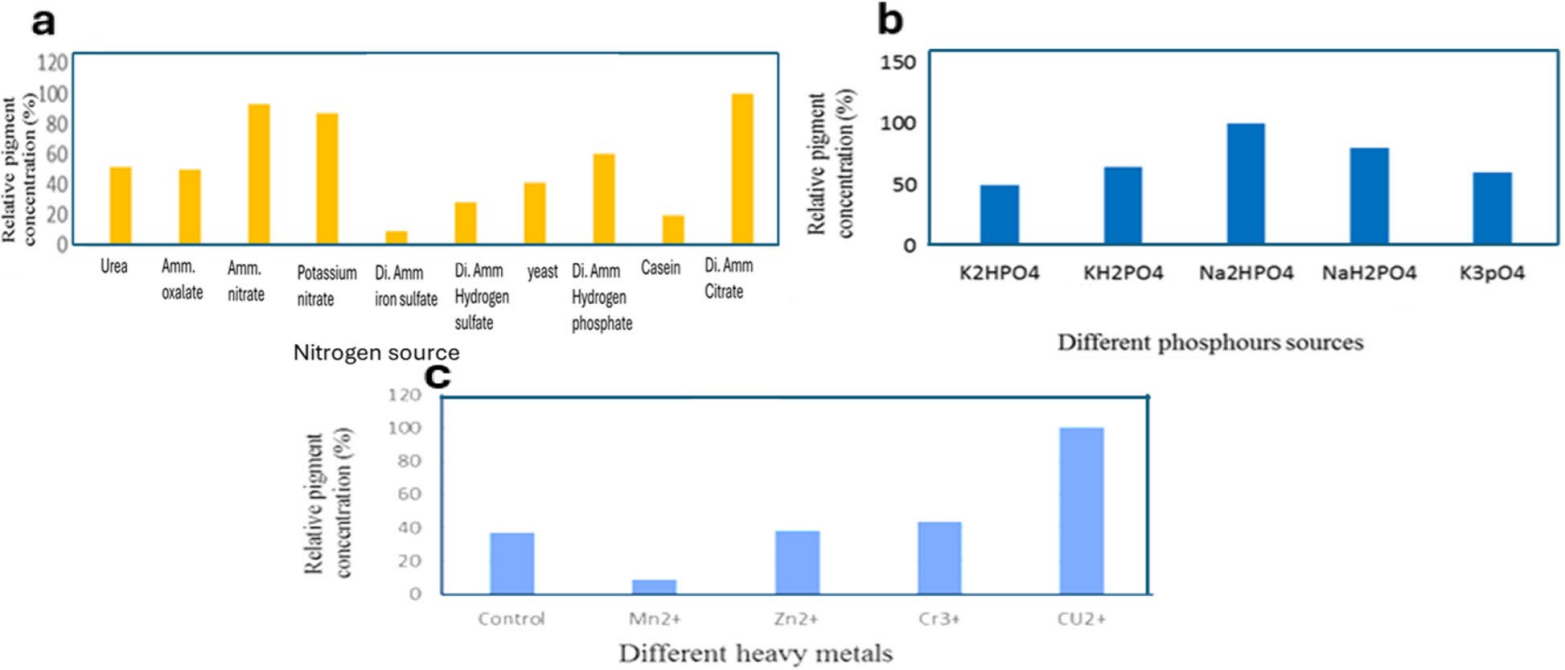
**a** Influence of inorganic nitrogen source, **b** Effect of phosphorous source, **c** Effect of metal ions on red pigment production by *S. phaeolivaceus* GH27

It is commonly recognized that nitrogen plays an essential role for the growth of all living organisms and that the availability of nitrogen sources in the growth medium has a significant impact on nitrogen metabolism. The consumption of the nitrogen source and the metabolism associated with a favorable metabolic way are both necessary for the formation of pigments [35]. Previous studies have demonstrated that the impact of a nitrogen source on pigment production differs depending on the strain [34]. While utilizing one nitrogen source can promote pigment formation, employing a different nitrogen source could inhibit pigment production by the same isolate. For instance, diammonium citrate is more suitable than other inorganic nitrogen sources for more red pigment secretion in the growth medium inoculated with *S. phaeolivaceus* GH27 (Fig. 4a).

### Effect of phosphorous source on the red pigment production

Using dibasic sodium phosphate was the prefilled phosphate source among the five phosphorous sources examined for maximal red pigment production by *S. phaeolivaceus* GH27 in the growth medium. The lowest one, however, was sodium dihydrogen phosphate (Fig. 4b). El Sayed et al. (2023) had similar results [3].

### Effect of metal ion addition

Metal ions are very essential for biosynthesis pathways in cells as enzymes, coenzymes, and bioactive compound production. Data indicated that the Cu^++^ ion is very essential in the culture medium for red pigment production by *S. phaeolivaceus* GH27 (Fig. 4c). El Sayed et al. (2023) reported that the addition of Cr^3+^ ions enhanced green pigment production [3].

### Extraction of red pigment produced by *S. phaeolivaceus* GH27

In fact, the highest yield of red pigment extract is produced by using ethanol as an extracting solvent via a liquid–liquid extraction method that was found to be different from the reports by Abraham and Chauhan (2018) [16]. Thereafter, the recovered extract was vacuum dried at a lower pressure (Supplementary. 1). El Sayed et al. (2023) had similar results [3].

### Effect of heat treatment on red pigment stability produced by *S. phaeolivaceus* GH27

**Fig. 5:**
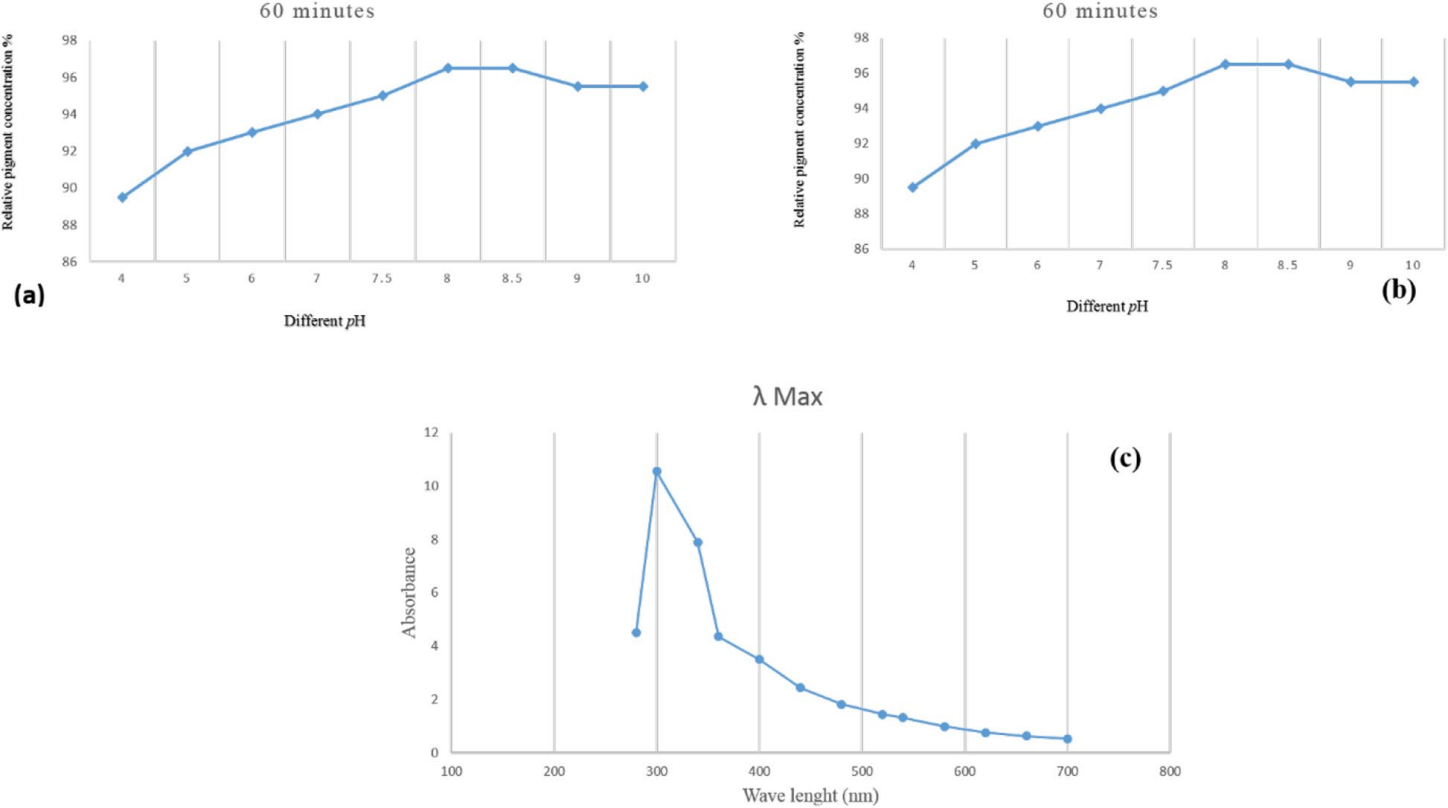
**a** Effect of heat treatment on the retention of extracted red pigment heated for one hour at different temperatures, **b** Effect different *p*H on stability of extracted red pigment, **c** The UV absorption spectrum of extracted red pigment produced by *S. phaeolivaceus* GH27

Heat treatment significantly influences the stability of red pigment.At 40 °C and 50 °C, no significant loss occurred in pigment content. Even by heating pigment extract at 60, 70, 80, 90, and 100 °C for one hour, retention of pigment is still as high as 98, 96.5, 95.5, 94.6, and 92.6% of the total pigment content, respectively. (Fig. 5a).

### Effect of pH on the stability of red pigment produced by *S. phaeolivaceus* GH27

From the results, it was observed that the red color of the extracts retained its high stability at alkaline* p*H (Fig. 5b).

### UV-absorption spectrum of extracted red pigment produced by *S. phaeolivaceus* GH27

The UV absorption spectrum of red pigment extract produced by *S. phaeolivaceus* GH27 is shown in Fig. 5c. Data indicated that the absorption maximum peak of the extract of red pigment was 280—340 nm, having a *λ*_max_ at 300 nm. However, Kazi et al. 2022 have reported a *λ*_max_ 247 for the produced pigment from *Streptomyces* species on full UV scanning [1]. On the other hand, Selim et al. (2008) have demonstrated a *λ*_max_ 520 for roselle pigments [47].

### GC/MS characterization of red pigment produced by *S. phaeolivaceus* GH27

**Fig. 6:**
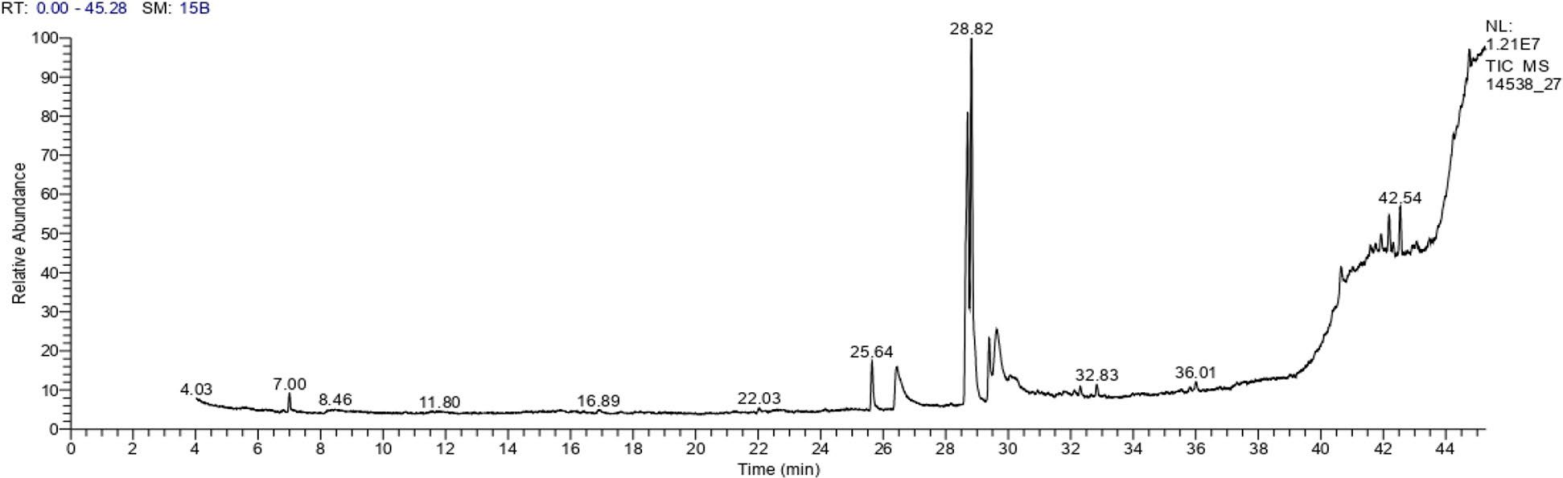
TIC chromatogram for the extracted red pigment from *S. phaeolivaceus* GH27 isolate

The most powerful analytical technique for organic analysis is gas chromatography-mass spectrometry (GC–MS). Because of its selectivity, sensitivity, and specificity, it is now widely used in environmental sample analysis. The different constituents are identified using GC–MS by comparing the spectrum of unknown compounds with the compounds stored in NIST (the National Institute of Standards and Technology Mass Spectral database) and the WILEY library of GC–MS [48] (Figure 6).

As illustrated in Table 1, about 8 compounds were identified from the pigment analysis by GC–MS and accounted for 76.43% of the separated compounds. It is clear that the main component (26.41%) was linolenic acid, methyl ester, with a slight difference from the second constituent (oleic acid, methyl ester (25.25%), then followed by palmitic acid (7.00%), oleic acid (6.82%), stearic acid, methyl ester (4.42%), palmitic acid, methyl ester (4.08%), oxiraneundecanoic acid, 3-pentyl-, methyl ester, cis (1.12%), and hexadecane (1.33%). It is possible to conclude that the pigment consists mainly of several fatty acids and only one alkane hydrocarbon (Hexadecane). The result is agreed with those obtained by El Sayed et al. (2023) on the dark green pigment [3], as they found lactic acid (19.72%) as the main component, followed by methylbutanoic acid (11.82%), carbamic acid (7.80%), ethylene (6.35%), and other different fatty acids. Because humans are unable to synthesize omega-6 fatty acids, such as linoleic acid, they are known to be essential fatty acids. In the body, omega-6 fatty acids play two major roles: first, they influence membrane function as structural components of membranes; second, they function as precursors of eicosanoids, which regulate inflammatory responses, vascular tone, renal, and pulmonary function [49]. Also, it has been found that linoleic acid has anticancer properties [50]. Oleic acid is one of the omega-9 fatty acids. It is both produced by the body and present in food. Oleic acid affects human health and disease in different ways. In addition to helping wounds heal, several studies have indicated that it may help prevent cancer, autoimmune, and inflammatory diseases [51]. As Carrillo et al. (2012) reported, one anti-inflammatory fatty acid that contributes to the activation of various immune-competent cell pathways is oleic acid [52]. It may also play a part in the development of new treatment modalities in the future for infections, inflammatory, immunological, cardiovascular, or skin repair [51].

**Table 1:** The relative percentage of the main constituents of produced red pigment

R.T	Area %	MF	MW	Compounds
7.00	1.33	C16H34	226	Hexadecane
25.64	4.08	C17H34O2	270	Palmitic acid, methyl ester
26.43	7.00	C16H32O2	256	Palmitic acid
28.70	26.41	C19H32O2	292	Linolenic acid, methyl ester
28.82	25.25	C19H36O2	296	Oleic acid, methyl ester
29.38	4.42	C19H38O2	298	Stearic acid, methyl ester
29.63	6.82	C18H34O2	282	Oleic Acid
32.83	1.12	C19H36O3	312	Oxiraneundecanoic acid, 3-pentyl-, methyl ester, cis

### Printing of cotton, wool, and polyester fabrics with extract red pigment

#### Effect of mordants on the color measurements

Using the natural red pigment produced by *S. phaeolivaceus* strain GH27 to dye cotton, wool, and polyester fabrics can be an intriguing application. Pre-treating the fabrics with tannic acid as a mordant is a commonly used method to enhance the absorption of natural dyes [53]. Tannic acid acts as a mordant by binding with the fabric fibers and creating sites for the dye molecules to attach, which helps improve color fastness and overall dye uptake. This pre-treatment is especially useful for natural dyes like those produced by *S. phaeolivaceus* strain GH27. When using this natural red pigment to dye fabrics such as cotton, wool, and polyester after pretreatment with tannic acid, it is important to consider factors such as *p*H levels during dyeing, temperature control, and post-dye finishing processes. Additionally, thorough washing and light fastness tests should be conducted on dyed fabrics to ensure their durability. The application of this natural red pigment in fabric dyeing offers potential for eco-friendly and sustainable textile coloration while leveraging existing knowledge on pre-treatments like mordants to maximize its effectiveness. Testing different concentrations of the pigment in combination with various mordants could lead to optimized results in terms of color intensity and wash-fastness.

#### Effect of pigment concentration

The concentration of red pigment used in the printing process has a significant impact on the dye uptake and color strength (K/S values) of wool, cotton, and polyester fabrics. The study found that increasing the dye concentration led to substantial improvement in the color strength of dyed wool, cotton, and polyester fabrics. Specifically, as the dye concentration increased from 0.5% to 2% on the mass of fabric (o.m.f.), there was a notable enhancement in the color strength (K/S values) of dyed wool samples. This suggests that higher concentrations of red pigment resulted in improved dye uptake and saturation of adsorption on the fiber. The findings imply that for achieving optimal color strength in wool fabric dyeing, higher concentrations of red pigment are favorable until reaching saturation point at 2%. It is important to note that similar studies should be conducted for cotton and polyester fabrics to determine their optimal dye concentration levels for achieving the desired color strength.

#### The effect of pH on the printing paste

**Fig. 7:**
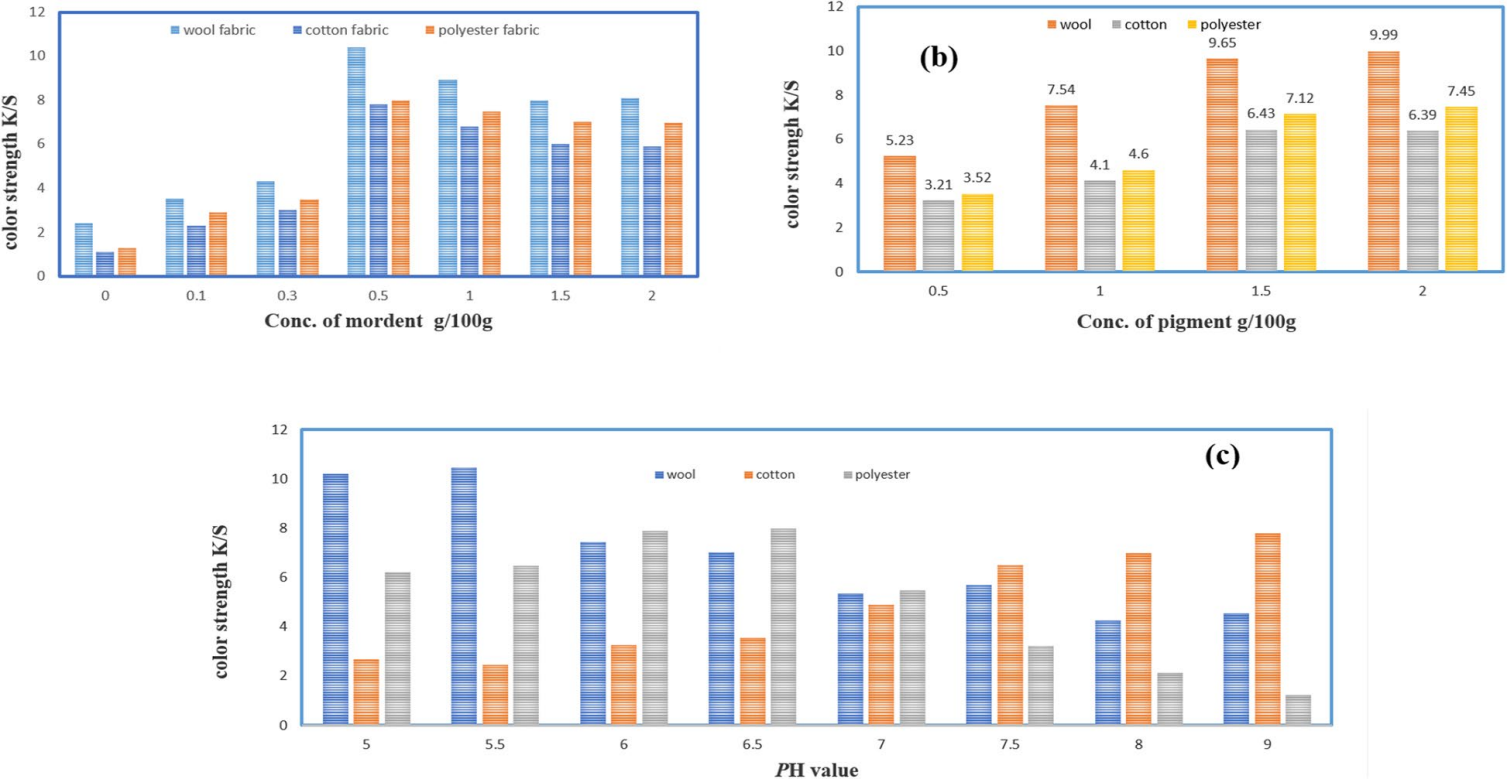
**a** Effect of concentration of mordant, **b** Effect of dye concentration, **c** Effect of *p*H value on (K/S) values of printing wool, cotton, and polyester fabrics with red pigment

The printing paste's *p*H had a crucial effect on the rate of dye fixation and can have a significant impact on the color strength (K/S values) of printed wool, cotton, and polyester substrates with red pigment. The study indicated that varying the *p*H of the printing paste had different effects on each type of fabric. For wool, reducing the *p*H of the printing paste resulted in an increased dye fixation rate due to higher dye concentration and ammonium ion sites at lower *p*H values [54]. This led to an optimum K/S value at a *p*H of 5.5. On pretreated cotton with tannic acid, an optimum K/S value was achieved at a higher *p*H level of 9. This indicates that for cotton prints, a more alkaline environment was favorable for achieving optimal color strength. In contrast, polyester fabrics showed an optimal K/S value at a slightly acidic *p*H level of 6.5. The study revealed that for polyester substrates with red pigment, maintaining a neutral to slightly acidic environment was most effective for achieving maximum color strength. Overall, these findings emphasize the importance of considering fabric type-specific optimal *p*H levels when aiming to achieve desired color strength and dye fixation rates in natural dyeing processes using red pigment and tannic acid as pretreatment agents on textile materials like wool and cotton or even synthetic fibers like polyester (Figure 7).

#### Fastness properties

The natural red pigment produced by *S. phaeolivaceus* strain GH27 can be used to dye cotton, wool, and polyester fabrics. The fastness characteristics of dyed fabrics refer to how well the color holds up over time and with exposure to things like washing, light, and rubbing [55]. Table 2 shows the fastness properties of the printed cotton, wool, and polyester fabrics with extracted red pigment. The printed fabrics have excellent fastness properties with red pigment. Light fastness qualities were very good for all printed fabrics, illustrating the pigment's capability for higher-quality fabrics printed because of chemical bonds that formed between the fabrics and pigment molecules. Table 2 shows that color strength demonstrated by (K/S) values had higher values for wool fabrics printed with pigment than cotton and polyester printed fabrics. Colorfastness for washing evaluates how well the color holds up when the fabric is washed or exposed to water. Light fastness measures how resistant the color is to fading when exposed to light. Rubbing or abrasion fastness assesses how well the color withstands rubbing or friction. These tests would provide information on how well the red pigment adheres to and withstands various conditions on cotton, wool, and polyester fabrics.

**Table 2:** Color strength (K/S) fastness characteristics of printed cotton, wool and polyester fabrics at ideal condition of mordant 0.5 g/100gm, concentration of pigment 2 g/100 g and PH value

Fabric samples	Washing fastness		Rubbing fastness		Perspiration fastness				Light fastness
					Acidic		Alkaline		
	St	Alt	Dry	Wet	St	Alt	St	Alt	
wool	4	4	4–5	4	4–5	4–5	4	4–5	5–6
	4–5	4	4–5	4–5	4–5	4	4–5	4–5	6
	4–5	4–5	5	5	4–5	4–5	4	4–5	6–7
	5	4–5	5	5	4–5	5	4–5	5	6–7
Cotton	3–4	3–4	4	3–4	4–5	4–5	4–5	4–5	5
	4	3–4	4	3–4	4–5	4–5	4–5	4–5	5–6
	3–4	4	4–5	4	5	5	4–5	4–5	5–6
	4–5	4	4–5	4	5	5	4–5	4–5	4–6
polyester	4	3–4	4	3–4	4–5	4–5	4–5	4–5	5–6
	4–5	3–4	4–5	3–4	4–5	4–5	4–5	4	5–6
	4–5	4	4–5	4	5	5	4–5	4–5	6
	4–5	4	4–5	4	5	5	5	5	6

### Antimicrobial evaluation of the treated fabrics

**Fig. 8:**
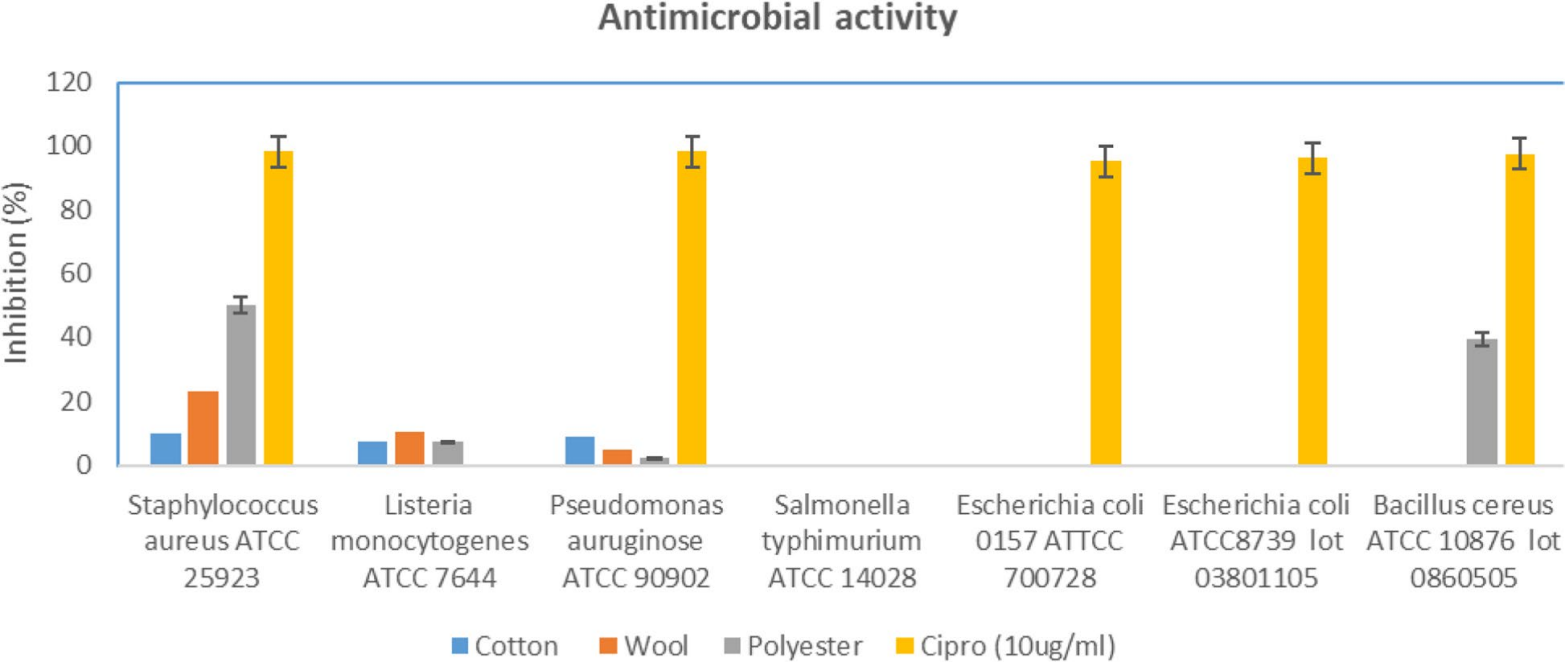
Antimicrobial evaluation of the treated fabrics (cotton, wool and polyester)

The efficacy of treated cotton, wool, and polyester samples in inhibiting the growth of different pathogens exhibited different levels of efficiency. The cotton sample exhibited a moderate level of inhibition against *Staphylococcus aureus* (10%), *Listeria monocytogenes* (7.26%), and *Pseudomonas aeruginosa* (9%), but did not demonstrate any action against *Salmonella typhimurium*, *Escherichia coli* O157, *Escherichia coli* ATCC 8739, or *Bacillus cereus*. Wool has shown a somewhat greater level of antibacterial activity, namely against *Staphylococcus aureus* (23.02%) and *Listeria monocytogenes* (10.23%). Its effectiveness against *Pseudomonas aeruginosa* was modest (5%), and it had no impact on the other infections. The sample treated with polyester exhibited the highest level of inhibition against *Staphylococcus aureus* (50.03%) and *Bacillus cereus* (39.49%), displaying moderate activity against *Listeria monocytogenes* (7.31%) and minimal activity against *Pseudomonas aeruginosa* (2.13%). However, it had no effect on the other pathogens that were tested. Ciprofloxacin, used as a reference standard (Fig. 8).

### In silico predictions of ADME-related physicochemical properties and toxicity prediction

The Swiss ADME platform [56] was utilized to measure the physicochemical parameters of linolenic acid and methyl ester that are relevant to ADME. The calculations rely on a characterization of the generated molecule based on the drug-likeness criteria. Consequently, the compound passed the Lipinski test with just one violation (MLOGP > 4.15), but it had two violations for the Veber test (WLOGP > 5.6) and the Ghose test (Rotors > 10). Its bioavailability (Supplementary 2) is 0.55.

**Fig. 9:**
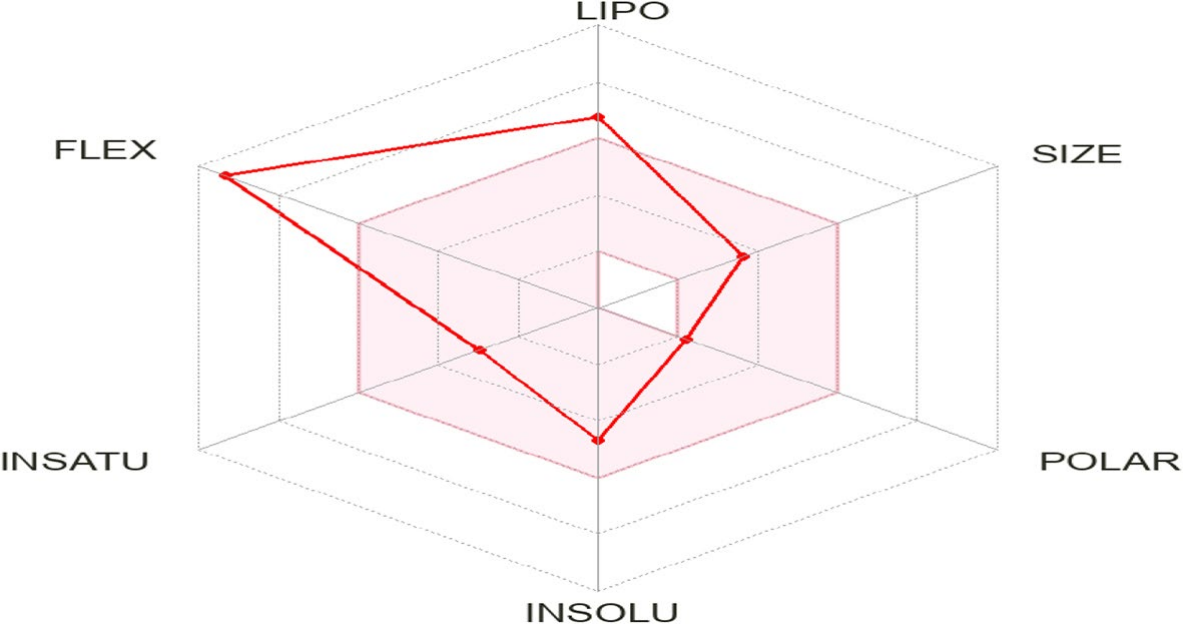
Bioavailability Radar plot of the abundant compound. The pink area shows the optimal range for each property (Lipophilicity: XLOGP3 between − 0.7 and + 5.0, size: MW between 150 and 500 g/mol, polarity: TPSA between 20 and 130 Å^2^, solubility: log *S* not higher than 6, saturation: fraction of carbons in the sp.^3^ hybridization not less than 0.25, and flexibility: no more than 9 rotatable bonds)

**Fig. 10:**
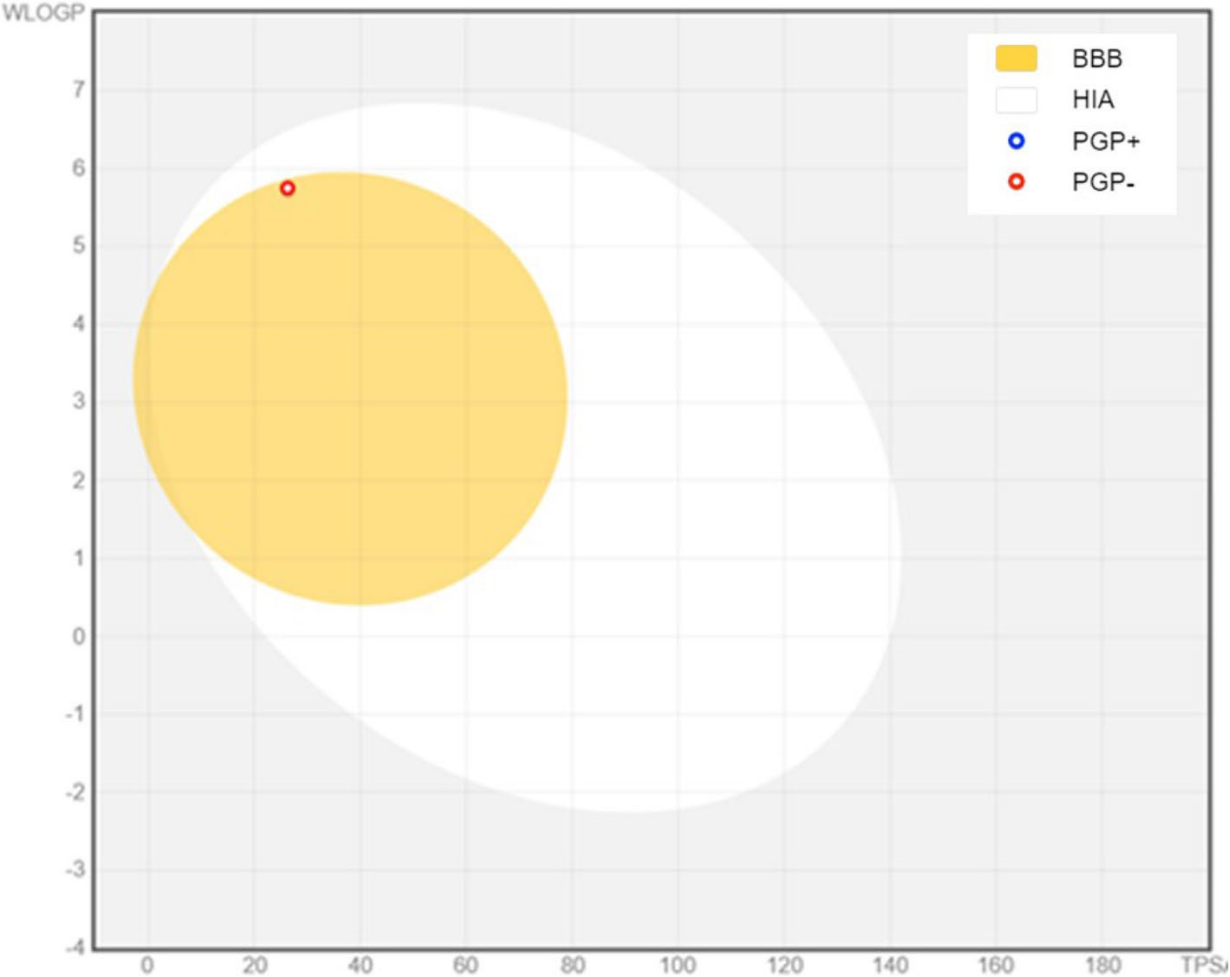
BOILED-Egg plot for most abundant compound. The yellow zone (yolk) is for highly possible BBB permeability, while the white region (GI) is for highly probable HIA (GI) absorption

Additionally, the drug-likeness was quickly determined by analyzing the bioavailability radar plot (Fig. 9), which relies on six physicochemical properties: size, lipophilicity, polarity, flexibility, solubility, and saturation. The pink region in the graph represents the ideal range of amounts for each parameter. The developed figure revealed that the substance exhibited the optimal range (pink zone) for all parameters except for flexibility. Lipophilicity, a physicochemical measure that indicates a compound's permeability across a cell membrane, is another significant one [57, 58]. Log Po/w values for the studied chemical were less than 5, specifically 3.60, indicating good permeability and absorption across the cell membrane. Furthermore, the solubility of a substance is a crucial factor that affects its absorption in almost every formulation procedure [56]. The compound exhibits solubility according to the ESOL topological model. The compound fails to meet the rule of three (RO3) for determining medicinal chemistry and lead toxicity due to two violations: XLOGP3 surpassing 3.5 and Rotors exceeding 7. With a synthetic accessibility score (SAscore) of 3.10, calculated based on complexity penalties and fragment similarity, the chemical exhibited a moderate level of synthetic accessibility. Table 3 [56] presents the compound pharmacokinetic parameters estimated using the vector machine algorithm (SVM) model. The compound exhibits selective inhibition of the isoenzymes CYP1A2 and CYP2C9. Figure 10 displays the BOILED-Egg model, which measures the brain or intestinal D permeation using the WLOGP versus TPSA method. The chemical has a very efficient rate of gastrointestinal absorption (GI) in humans. The compounds exhibit a blood–brain barrier (BBB) permeability (TPSA 26.30 Å^2^) and are non-phosphogalactone (PGP-) substrates (red dots), indicating their impact on the central nervous system (CNS). The compound exhibited a logarithmic (Kp) permeance of -3.62 cm/s. A higher negative log Kp value indicates lower skin permeability of the compound (Fig. 10). The methods for predicting the skin permeability coefficient (Kp) of the synthesized chemical were elucidated in references [58, 59].

**Table 3:** Pharmacokinetics properties of the most abundant compound

GI absorption	High
BBB permeant	Yes
P-gp substrate	No
CYP1A2 inhibitor	Yes
CYP2C19 inhibitor	No
CYP2C9 inhibitor	Yes
CYP2D6 inhibitor	No
CYP3A4 inhibitor	No
Log Kp (skin permeation)	-3.62 cm/s

## Conclusion

Streptomycete isolate *S. phaeolivaceus* GH27 was identified via 16S rRNA gene sequencing. The isolate produced a stable red pigment optimized under specific conditions and extracted efficiently in ethanol. The pigment demonstrated thermal and pH stability, with key components identified as linolenic and oleic acid methyl esters. When used for fabric printing, the pigment showed strong color fastness and eco-friendly properties. Antimicrobial testing revealed effective inhibition of *Staphylococcus aureus* and *Bacillus cereus*, particularly on polyester. ADME analysis suggested favorable drug-like properties with good absorption and permeability but moderate synthetic accessibility.

## Supplementary Information

Supplementary Material 1.

## Data Availability

The datasets generated and/or analysed during the current study are available in the NCBI repository, https://www.ncbi.nlm.nih.gov/nuccore/OQ145635.1/ with accession number OQ145635.
