# Supplementary material for: Improving natural red pigment production by Streptomyces phaeolivaceus strain GH27 for functionalization of textiles with in silico ADME prediction
Source: BMC Microbiol. 2025 Jan 13;25:19. doi: 10.1186/s12866-024-03697-4 (PMC11726976; doi:10.1186/s12866-024-03697-4)
Supplement: Supplementary file 1 — Supplementary Material 1. [file 12866_2024_3697_MOESM1_ESM.docx]

| 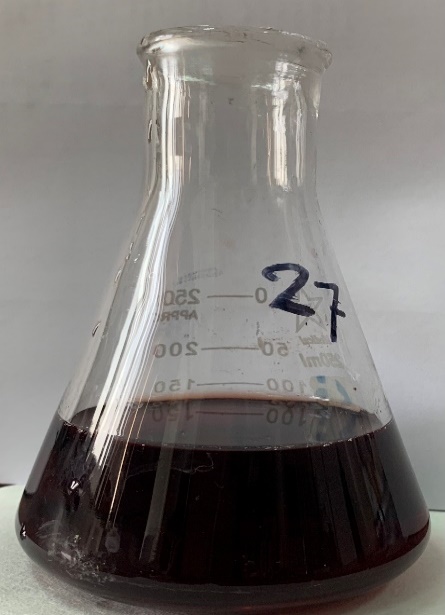  **(a)** | **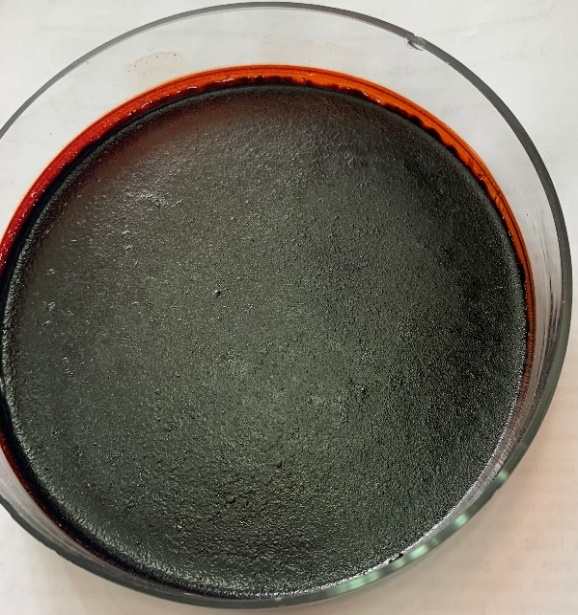**  **(b)** |
| --- | --- |
| Supplementary 1. (a). Pigment extracted by ethanol solvent; (b). Dried pigment after ethanol extraction | |

Supplementary 2. ADME physicochemical properties of the most abundant compound

| **Category** | **Property** | **Value** |
| --- | --- | --- |
| **Physicochemical Properties** | Formula | C19H32O2 |
|  | Molecular weight | 292.46 g/mol |
|  | Num. heavy atoms | 21 |
|  | Num. arom. heavy atoms | 0 |
|  | Fraction Csp3 | 0.63 |
|  | Num. rotatable bonds | 14 |
|  | Num. H-bond acceptors | 2 |
|  | Num. H-bond donors | 0 |
|  | Molar Refractivity | 93.31 |
|  | TPSA | 26.30 Å² |
| **Lipophilicity** | Log Po/w (iLOGP) | 4.94 |
|  | Log Po/w (XLOGP3) | 6.29 |
|  | Log Po/w (WLOGP) | 5.75 |
|  | Log Po/w (MLOGP) | 4.61 |
|  | Log Po/w (SILICOS-IT) | 6.18 |
|  | Consensus Log Po/w | 5.55 |
| **Water Solubility** | Log S (ESOL) | -4.69 |
|  | Solubility (ESOL) | 5.94e-03 mg/ml ; 2.03e-05 mol/l |
|  | Class (ESOL) | Moderately soluble |
|  | Log S (Ali) | -6.63 |
|  | Solubility (Ali) | 6.85e-05 mg/ml ; 2.34e-07 mol/l |
|  | Class (Ali) | Poorly soluble |
|  | Log S (SILICOS-IT) | -4.65 |
|  | Solubility (SILICOS-IT) | 6.49e-03 mg/ml ; 2.22e-05 mol/l |
|  | Class (SILICOS-IT) | Moderately soluble |
| **Pharmacokinetics** | GI absorption | High |
|  | BBB permeant | Yes |
|  | P-gp substrate | No |
|  | CYP1A2 inhibitor | Yes |
|  | CYP2C19 inhibitor | No |
|  | CYP2C9 inhibitor | Yes |
|  | CYP2D6 inhibitor | No |
|  | CYP3A4 inhibitor | No |
|  | Log Kp (skin permeation) | -3.62 cm/s |
| **Druglikeness** | Lipinski | Yes; 1 violation: MLOGP>4.15 |
|  | Ghose | No; 1 violation: WLOGP>5.6 |
|  | Veber | No; 1 violation: Rotors>10 |
|  | Egan | Yes |
|  | Muegge | No; 1 violation: XLOGP3>5 |
|  | Bioavailability Score | 0.55 |
| **Medicinal Chemistry** | PAINS | 0 alert |
|  | Brenk | 1 alert: isolated_alkene |
|  | Leadlikeness | No; 2 violations: Rotors>7, XLOGP3>3.5 |
|  | Synthetic accessibility | 3.10 |
